# High-Throughput Metabolomics Method for Discovering Metabolic Biomarkers and Pathways to Reveal Effects and Molecular Mechanism of Ethanol Extract From *Epimedium* Against Osteoporosis

**DOI:** 10.3389/fphar.2020.01318

**Published:** 2020-08-27

**Authors:** Jun-feng Zhao, Jian-yu Xu, Yi-er Xu, Shui-lin Chen, Yan-xing Guo, Quan-yang Gao, Gui-cai Sun

**Affiliations:** ^1^ The Manual Orthopaedics, Henan Province Luoyang Orthopedic Thraumatological Hospital (Henan Provincal Orthopedic Hospital), Luoyang, China; ^2^ The Tumor Hospital of Harbin Medical University, The Department of Radiation Oncology, The Affiliated Tumour Hospital of Harbin Medical University, Harbin, China; ^3^ The Research & Development Center of Harbin Pharmaceutical Group, The Laboratory of Pharmacology Quality Inspection & Pilotscale Experiment Workshop, Harbin, China; ^4^ The Department of Orthopaedics, The Fourth Hospital Attached to Nanchang University, Nanchang, China; ^5^ The First Affiliated Hospital of Nanchang University, Orthopaedics, Nanchang University, Nanchang, China

**Keywords:** metabolomics, biomarker, metabolism, pathway, molecular mechanism, osteoporosis

## Abstract

Metabolomics is an effective strategy to explore the molecular mechanism of herbal medicine. Epimedium, a traditional Chinese herb from the Epimedium brevicornu Maxim., has a therapeutic effect on osteoporosis (OP), however the molecular mechanism of the anti-OP effect is uncle\ar. Therefore, we investigated the pharmacological effect and action mechanism of ethanol extract of epimedium (Ext-epi) onOP rat model. The serum of OP rats was analyzed utilized UPLC-Q-TOF/MS metabolomics, and the potential biomarkers were screened and identified using multivariate data analysis systems and network databases. To further appraise the influence of Ext-epi on biological markers and metabolic pathways, and reveal the potential mechanism of Ext-epi on OP treatment. The results showed that 46 potential biomarkers were screened out and after intervention with Ext-epi extracts solution, 16 potential biomarkers were significantly recalled. Further pathway experiments showed that key pathway analysis include sarachidonic acid metabolism, glycerolphospholipid metabolism as potential targets which is related with the efficacy of Ext-epi protect against OP. These results explain the correlation between metabolites and molecular mechanisms, which is of great significance for understanding the intervention of Ext-epi on OP. In short, based on UPLC-Q-TOF/MS metabolomics may provide effective strategies for understanding the pathogenesis of diseases and evaluating the intervention effect of natural products.

## Introduction

Osteoporosis (OP) is a systemic bone metabolic disease that seriously puzzles the elderly (especially postmenopausal women). With the development of social economy and the improvement of living standards, the aging trend is becoming more and more obvious, and the incidence of OP is also gradually increasing. World health organization (WHO) regards OP as the second most fatal health care problem after cardiovascular diseases (Liu et al., 2020). Bone mineral density (BMD) is currently the main index for OP disease assessment and fracture prediction (Diab and Watts, 2013). However, BMD cannot truly reflect the changes of OP due to its low sensitivity and slow changes in the course of the disease. At present, there is no index that can accurately and objectively evaluate the prognosis of OP. The loss of bone is caused by imbalance of bone formation and bone resorption by osteoclasts (Gundala et al., 2018). Most OP treatments aim at adjusting this imbalance. The treatment plan of OP mainly includes the following points: strengthening exercise, enhancing the nutrition supplement, preventing wrestling to prevent fracture, and drug treatment. At present, drugs for treating OP are mainly bisphosphate, salmon calcitonin, calcium preparations, etc (He et al., 2010). These therapeutic drugs basically belong to the single-component and single-target treatment mode, which is difficult to adapt to the comprehensive treatment of chronic metabolic diseases caused by OP and other reasons. Serious adverse reactions and drug dependence may occur after long-term administration, which brings great pain to patients. In recent years, with the continuous development of traditional Chinese medicine, single drug and compound preparations are consistent with the mechanism of chronic metabolic diseases with their concept of multi-component and multi-target comprehensive treatment, and their adverse reactions and drug dependence are relatively low. Therefore, it has become a trend to prevent and treat OP with herbal medicine and its extracts (Zhang A. et al., 2013).

Metabolomics technology combine with high-throughput, high-resolution analysis techniques, and multivariate data analysis method to explore the characteristics and rules of metabolic level changes throughout the life activity (Zhang et al., 2014; Liang et al., 2015), thereby establish the relationship between changes in metabolite content and changes in biological phenotypes relationship, in order to reveal the metabolic mechanism of disease occurrence and development (Zhang A. H. et al., 2013; Liang et al., 2017a), and provide important information for further exploration of therapeutic targets. In recent years, metabolomics technology has played a prominent role in the diagnosis of diseases and the discovery of biomarkers, providing a powerful means for the discovery of new drugs and the excavation of disease treatment targets (Liang et al., 2016a; Liang et al., 2016b). Researchers found that many metabolic pathways had changed significantly before pathological changes occurred in OP (Zhang et al., 2019). A LC/MS combined with multivariate date analysis has been performed a metabolomics analysis on the plasma of ovariectomized osteoporosis model rats. The results characterized 18 differential metabolites and 13 related pathways include the metabolism of arachidonic acid and glycerophospholipids, etc.

*Epimedium* is a clinically effective Chinese herbal medicine that has been documented in previous books (Watts and Diab, 2010). It was first published in <Shennong Materia Medica>, and origin from the *Epimedium brevicornu* Maxim. The whole plant is used as medicine (Wu et al., 2018). According to records, Epimedium has the main effects of tonifying kidney, strengthening yang, dispelling wind and removing dampness (Wu et al., 2008). Its main active ingredients include icariin and total flavonoids. Modern pharmacological studies have shown that Epimedium has positive therapeutic effects on immune system, reproductive system, cardiovascular, and cerebrovascular systems, neuroendocrine system, bone metabolism, and anti-tumor. Studies have found that *Epimedium* contains 77 active ingredients with estrogen-like effects, 23 of which are related to anti-OP therapeutic effects (Wu et al., 2003). Flavonoids from *Epimedium* have the highest effect on OP osteoblast differentiation, which proves that they are effective components against OP. In this study, ultra-high performance liquid chromatography combined with quadrupole time-of-flight mass spectrometry (UPLC-Q-TOF/MS) technology was used to analyze serum of healthy rats, OP model rats and rats interfere with different doses of Ext-epi. On the premise of characterizing potential biomarkers and metabolic pathways of OP, the therapeutic effect of Ext-epi on OP was evaluated. The aim is to find potential biomarkers of OP and potential targets and related pathways of Ext-epi for its protective effect, and to provide a basis for clarifying the mechanism of action of Ext-epi in treating OP.

## Experiment

### Drugs and Reagents

Acetonitrile and methanol were provided by Merck (Merck. Germany). Leucine-enkephalin was purchased from Sigma-Aldrich Company (Shanghai, China). Formic acid was bought from Dikma Technologies Company (Beijing, China). Distilled water was acquired from China Watsons Food Co., Ltd. (Guangzhou, China). Pentobarbital sodium was acquired from Merck Pharmaceutical Biotechnology Co., Ltd., Germany, batch number 20180607 (Merck, Germany). Penicillin sodium was purchased from North China Pharmaceutical Co., Ltd., batch number F7086108 (Hebei, China). Osteocalcin, tumor necrosis factor-α(TNF-α), estradiol, and tartrate-resistant acid phosphatase (TRAP) Elisa kit were all bought from Nanjing Jiancheng Biotechnology Co., Ltd. (Nanjing, China). All reagents used are chromatographic grade.

### Preparation of Epimedium Solution

Epimedium (*Epimedium brevicornu* Maxim) was supplied by Harbin Branch of *Tongrentang* Pharmaceutical Co., Ltd., and the samples of medicinal materials (voucher numbers: HY201807314) are placed in the Herbarium of Nanchang University. The determination of epimedin A, epimedin B, epimedin C, and icariin has been established by HPLC (**Figure S1**). The reflux extraction with 6 time of 70% ethanol solution for 2 h, pouring out the extractive solution, extracting the residue for 2 h again, and combining the two extractive solutions, concentrated to 0.075 g/ml, and frozen. Freeze drying the extract to obtain loose powder, and sealing in a dryer for later use. When the extract is administered, precisely weigh freeze-dried powder and dissolve it in distilled water, and administer the solution with corresponding concentration to rats in different dosage groups.

### Animals and Sample Collection

Animal experiments were carried out in accordance with the animal experimental guidelines of Laboratory Animal Center of Nanchang University and approved by the Animal Ethics Committee. 50 clean Wister rats (female, 240–280g) were provided by the Laboratory Animal Center of Nanchang University, and these were raised in a standard environment (temperature: 20–25°C, humidity: 50%–65%, light/dark cycle alternating every 12 h). All rats were acclimatized for 7 days, during which they were fed with free drinking water and standard feed. After adapting to the environment, the rats were randomly divided into control group (sham operation group), model group, *Epimedium* extract low dose group (ZL-IL), *Epimedium* extract medium dose group (ZL-IM), and *Epimedium* extract high dose group (ZL-IH), groups of animals were balanced to ensure lowest variation in average weight. After one week of acclimatization, all animals were fasted and forbidden water for 12 h before operation, and anesthesia (100 mg/kg) was administered by intraperitoneal injection of pentobarbital sodium at a concentration of 3%. After anesthesia, the abdominal cavity of the rats was placed on the operating table upward, and the rat hair on the surface of the operating site was cut off. Preoperative disinfection was performed with iodophor and 75% alcohol. In the sham operation group, the bilateral incisions of the lumbar vertebrae were taken into the abdominal back under aseptic conditions, and the bilateral ovaries were just moved outside the body, placed in a sterile environment for 1 min, and then carefully reset. The model group and epimedium treatment group completely removed bilateral ovaries in the same way. Suture muscle and skin after gauze hemostasis. The suture site was disinfected again and 50,000 international units of penicillin sodium were given to prevent infection. 50,000 international units of penicillin sodium were continuously given once a day one week after operation. After 2 weeks, all rats began to receive drug therapy after wound healing. ZL-IL [75mg/(kg.d)]; ZL-IM [150mg/(kg.d)]; ZL-IH [225 mg/(kg.d)] groups were given *Epimedium* extract by gavage for 8 weeks, the control group and model group were given the same amount of distilled water. The dose is adjusted according to the weight of the rats every monday.

After 24 h, the last administration, and anesthesia (100mg/kg) was administered by intraperitoneal injection of pentobarbital sodium at a concentration of 3%. 5 ml of blood was taken from the abdominal aorta of all animals and allowed to stand for 30 min. 4°C, 3,000 rpm, centrifugation for 10min. Serum was taken and placed in a refrigerator at -80°C for standby. After taking blood, carefully remove the right femur along the direction of the rat joint, use scissors and gauze to remove the muscle and connective tissue attached to the femur, and place it in 75% ethanol for later use. The experimental procedures were approved by the Animal Care and Ethics Committee at Nanchang University and all experiments were performed in accordance with the declaration of Helsinki. The DEXA bone densitometer (GE, USA) was used to scan the right femur and the scan was performed in a small animal model. After scanning, the bone mineral content (BMC) and BMD of the whole femur were analyzed using GE software. The contents of osteocalcin (OC), TNF-a, estradiol (E_2_), and TRAP in rat serum were detected by enzyme-linked immunosorbent assay (Elisa), strictly check the contents of various indexes in serum according to the guiding principles.

### Serum Preparation

Remove rat serum from refrigerator and thaw in ice bath. Take 200μL serum sample and vortex it for 10s. Add 800 μl of methanol: acetonitrile mixed solution in equal proportion and vortex for 30 s to precipitate protein. Thermo Sorvall ST 16R cryogenic ultra-high speed centrifuge (Thermo Group, USA) was used to centrifuge at 4°Cand 13,000 rpm for 20 min. The supernatant was removed and dried with nitrogen in a 40°C water bath. After blow-drying, 160 μl of methanol was added to the residue for 60 s, and 40 μl of water was added for 60 s. the residue was centrifuged at 13,000 rpm at 4°Cfor 10 min. The supernatant was removed and filtered with a 0.22 μm filter membrane for UPLC-Q-TOF/MS analysis.

### UPLC/MS Analysis Conditions

Chromatographic conditions: Waters Acquity™ UPLC Ultra High Liquid Chromatograph (Waters, USA), UPLC-MS detection system with BEH C18 column (2.11, 100 mm, 1.7 μm, Waters). The column temperature is 40°C; Mobile phase A was Acetonitrile (0.1% formic acid) and mobile phase B was water (0.1%formic acid).Flow rate is 0.4mL/min. The gradient elution conditions are as followed: 0–4 min, 2%–40% A; 4–10 min, 40%–80% A; 10.0–13.0 min, 80%–90% A; 13.0–14.0 min, 90%–98% A; 14–15 min, 98%–2% A; 15–17 min, 2% A.

Mass spectrometry conditions: ESI^+^ and ESI^-^ capillary voltage: 1500V and 1300V. The remaining parameter settings are the same, sample cone voltage: 60V; desolvation gas temperature and flow: 350°C and 750 L/h; ion source temperature: 110°C; the flow rate is 20 L/h. Calibration solution injectionrate 100 μl/min, calibration frequency 15 s, the range is *m/z* 50–1,500 Da. The quality of leucineenkephalin was calibrated ([M+H]^+^=556.2771, [M-H]^-^=554.2615). All data were collected by MassLynx™ (V4.1, Waters Corporation, Milford, MA, USA) software.

### Multivariate Data Processing and Identification of Potential Biomarkers

All collected serum metabolic spectrum data were leaded into Progenesis QI software (Waters, USA) for data normalization and pretreatment. Then the processed data are imported into Ezinfo 3.0.3 (Waters Corporation, USA) for multivariate statistical analysis. Principal component analysis (PCA) was used to observe the clustering among groups and to observe whether there were significant differences in metabolism among different groups as a whole. In order to better reflect the metabolic differences between the two groups of blood, orthogonal partial least square discriminant analysis (OPLS-DA) can be further used for analysis. According to the confidence level of each ion point in the S-Plot and the selection variable (VIP) in OPLS-DA, the contribution of metabolites to classification is known. The importance of ions is judged by projecting the importance of VIP and combining the value of p in T-TEST algorithm. Finally, ions with VIP value greater than or equal to 1 and p value less than or equal to 0.05 are selected as potential biomarkers. Then the accurate Rt-*m/z* data are extracted by TIC chromatogram in Masslynx software, the accurate molecular weight of the compound is determined within a reasonable measurement error range, and the possible element composition and chemical formula of the compound are predicted by element composition module. After that, the accurate *m/z* value is used to search in online database (HMDB, METLIN) and the mass spectrum information is preliminary assessment of metabolite matching possibilities. Then, using the MS/MS fragment information provided by the Masslynx system, combining the possibility of mass spectrometry and chemical structure decomposition, the compound was verified to determine the final structure of potential biomarkers. Then the biological significance of biomarkers is analyzed through databases.

## Results

### The Biochemical Index Analysis Results

There were 10 rats in each group, and one rat died in each of ZL-IH and ZL-IM groups during the experiment. DEXA bone mineral density meter and Elisa reagent method were used to detect the femoral BMD and serum biochemical indexes in rats. As showed in **Figure S2**, compared with the sham-operation group, the BMC and BMD of the femur in the model group are significantly reduced (p<0.05). After treatment with different doses of Ext-epi, the BMC and BMD of the femur are significantly higher than model rats, among which the ZL-IL group has an extremely significant difference (p<0.01), the contents of OC, TNF-α, and TRAP in the serum of rats in the model group are higher than sham-operation group, and the contents of E2 decreased significantly (p<0.01). After intervention with Ext-epi, the contents of biochemical indexes of rats in each treatment group showed a tendency of callback, and TNF-α, TRAP, and E2 had significant differences (p<0.05), with specific content changes shown in **Table S1**.

### Analysis of Serum Metabolic Spectrum

**Figure 1:**
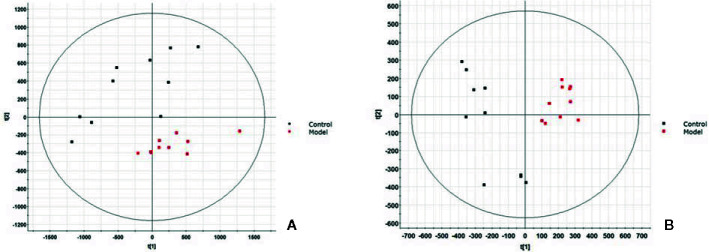
Score plot of serum profile of control group and model group scanned by principal component analysis (PCA) analysis. **(A)** Positive ion mode. **(B)** Negative ion mode.

**Figure 2:**
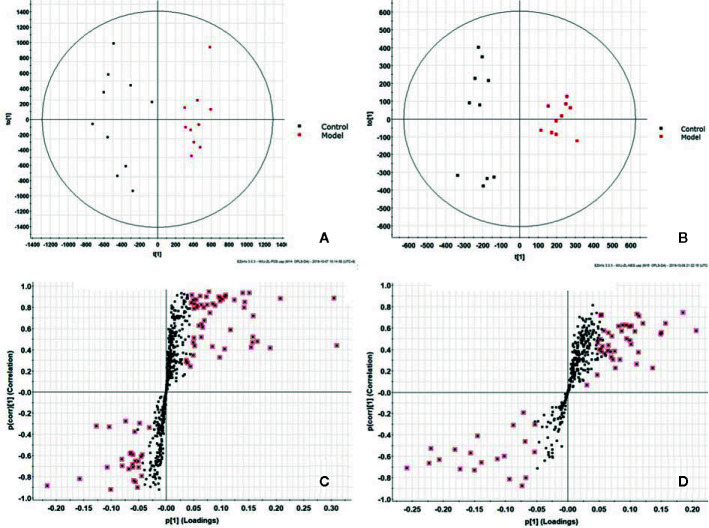
Orthogonal partial least square discriminant analysis (OPLS-DA) metabolic profiling of osteoporosis (OP) model: **(A)** score plot of serum profile of control group and model group scanned by OPLS-DA analysis in positive mode. **(B)** Score plot of serum profile of control group and model group scanned by OPLS-DA analysis in negative mode. **(C)** S-plot of serum profile of control group and model group scanned by OPLS-DA analysis in positive mode. **(D)** S-plot of serum profile of control group and model group scanned by OPLS-DA analysis in negative mode.

In this study, UPLC-Q-TOF/MS was used to perform a full scan of positive and negative ions on all serum samples under optimal conditions, and interspersed with one QC sample for every 10 samples taken to ensure the stability of the instrument operation. All the original data were imported intoMassLynxV4.1 software to obtain representative BPI profiles, the control and model group are shown in **Figures S3** and **S4**, respectively. It is found that the blood metabolic profiles are basically the same, but there are also some obvious differences. In order to better compare this difference and find out the potential differential metabolic molecules, this study uses the combination of non-targeted blood metabolomics and UPLC-Q-TOF/MS technology for analysis. The original data is imported into Progensis QI software, and each peak will be subjected to peak alignment, extraction, and peak generation processing. A total of 2,283 positive mode ions and 2,184 negative mode ions were detected and the parameters of the ion peak including *m/z*, Rt-time and relative intensity. Then transferred the ion information into Ezinfo3.0.3 software for multivariate data analysis. From the PCA score plot, we can see the model group was significantly separated from the control group, ESI+ as showed in **Figure 1A** and ESI- in **Figure 1B**. This indicates that the serum metabolism level of the model group rats has been significantly disturbed. Although there is still some overlap between the two groups, there has been a clear separation trend. In OPLS-DA diagram, two groups of significant separation can be clearly observed (**Figures 2A, B**).

### Identification of Potential Biomarkers

**Figure 3:**
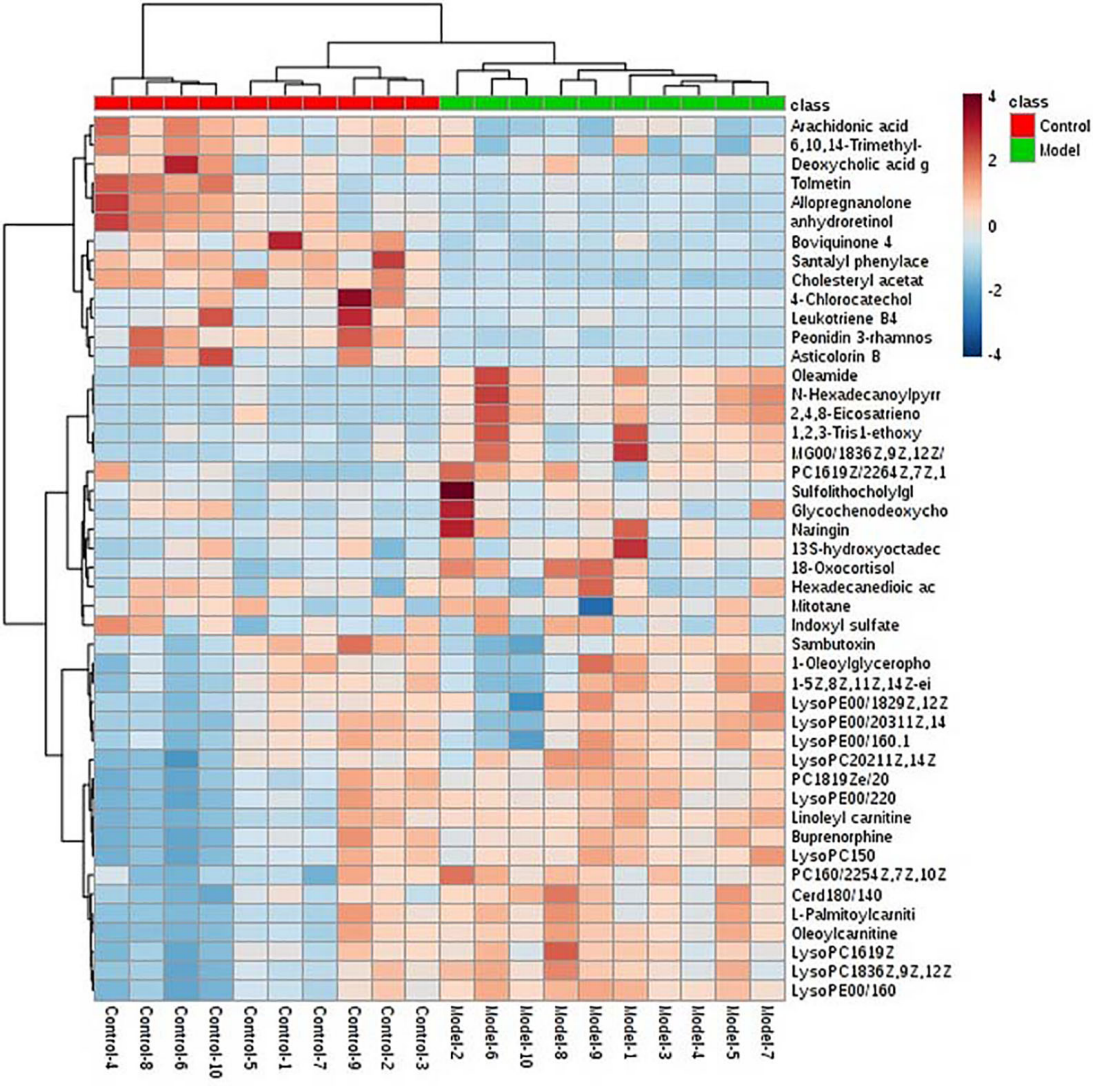
Heat map analysis of 46 differential metabolites in model group rats and control group rats. The degree of change in the mark with different colors, each row represents a single sample, and each column represents a metabolite. Top red represents the control group and the green represents the model group.

OPLS-DA is a statistical method of supervised discriminant analysis. In this method, Use OPLS-DA to analyze the group between the model group and the blank group to further evaluate the component separation. At the same time, the VIP value is used to determine the contribution rate of each ion to the separation between groups, so as to assist the screening of potential markers. In this study, OPLS-DA was carried out on the mass spectrum metabolic profiles of positive and negative ions. Score plot and 3D-score plotwere obtained as shown in **Figure 2A** (ESI+), **Figure 2B** (ESI-), and **Figure S5A** (ESI+), **Figure S5B** (ESI-), more significant differentiation changes between model group and control group were obtained. S-plot analysis charts are as show in **Figure 2C** (ESI+) and **Figure 2D**, (ESI-), the further away the ions from the distant point, the greater the contribution rate to the separation between groups. Combining with calculating the VIP (shown in **Figures S5C, D**), Themagnitude of the VIP value is directly proportional to the contribution rate of the degree of separation between the groups. We chose VIP > 1 and combined with ions with p < 0.05 in T-TEST calculation results as the next differential metabolite to be identified. Based on the accurate mass provided by the Q-TOF platform combined with HMDB, METLIN databases, preliminary identification of metabolites, and verification using MS/MS fragment ion information. Finally, 46 potential biomarkers were identified, 28 in ESI^+^, and 18 in ESI^-^ (**Table S2**). The levels of 35 metabolites in the model were significantly increased compared with the control group, and 11 metabolites decreased significantly. Based on the MetaboAnalyst (https://www.metaboanalyst.ca/) platform, a cluster heat map analysis of the content distribution of 46 metabolites among each other is shown in **Figure 3**, which clearly reveals the potential biological potential between Changes in the relative content of the markers.

### Metabolic Profiling Analysis

**Figure 4:**
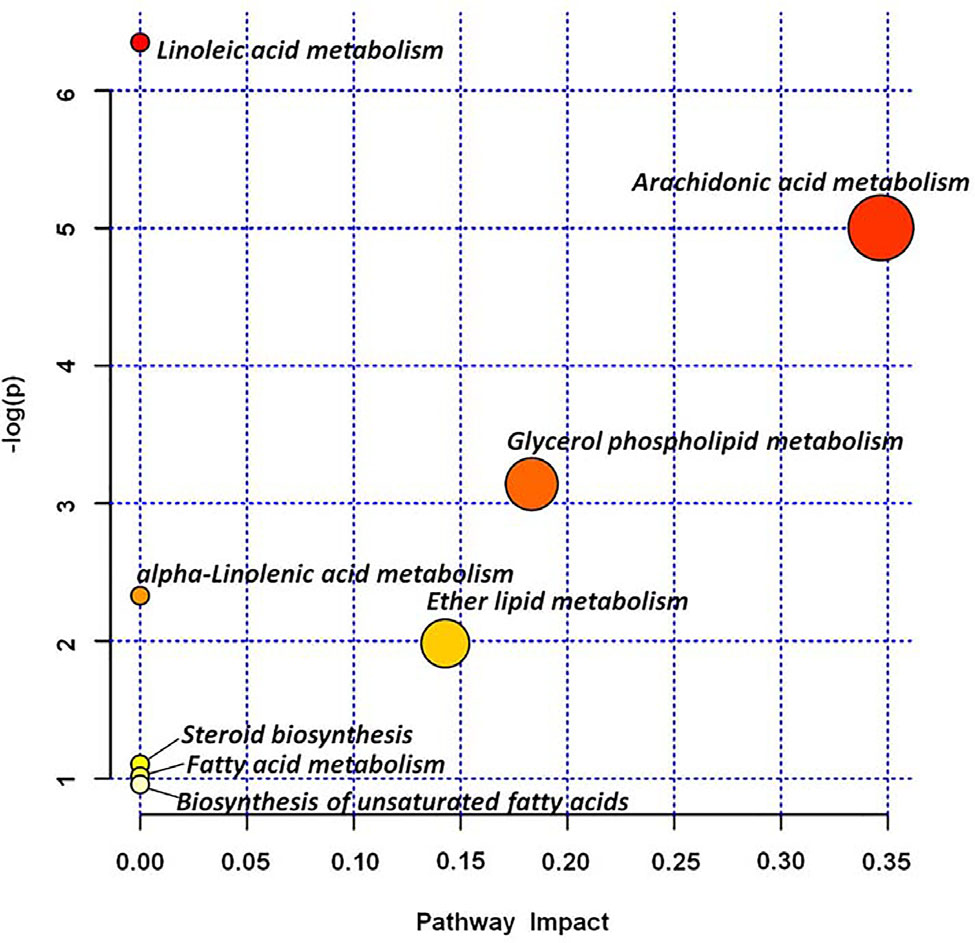
Metabolic pathway analysis of potential biomarkers in the serum generated using MetPA.

**Figure 5:**
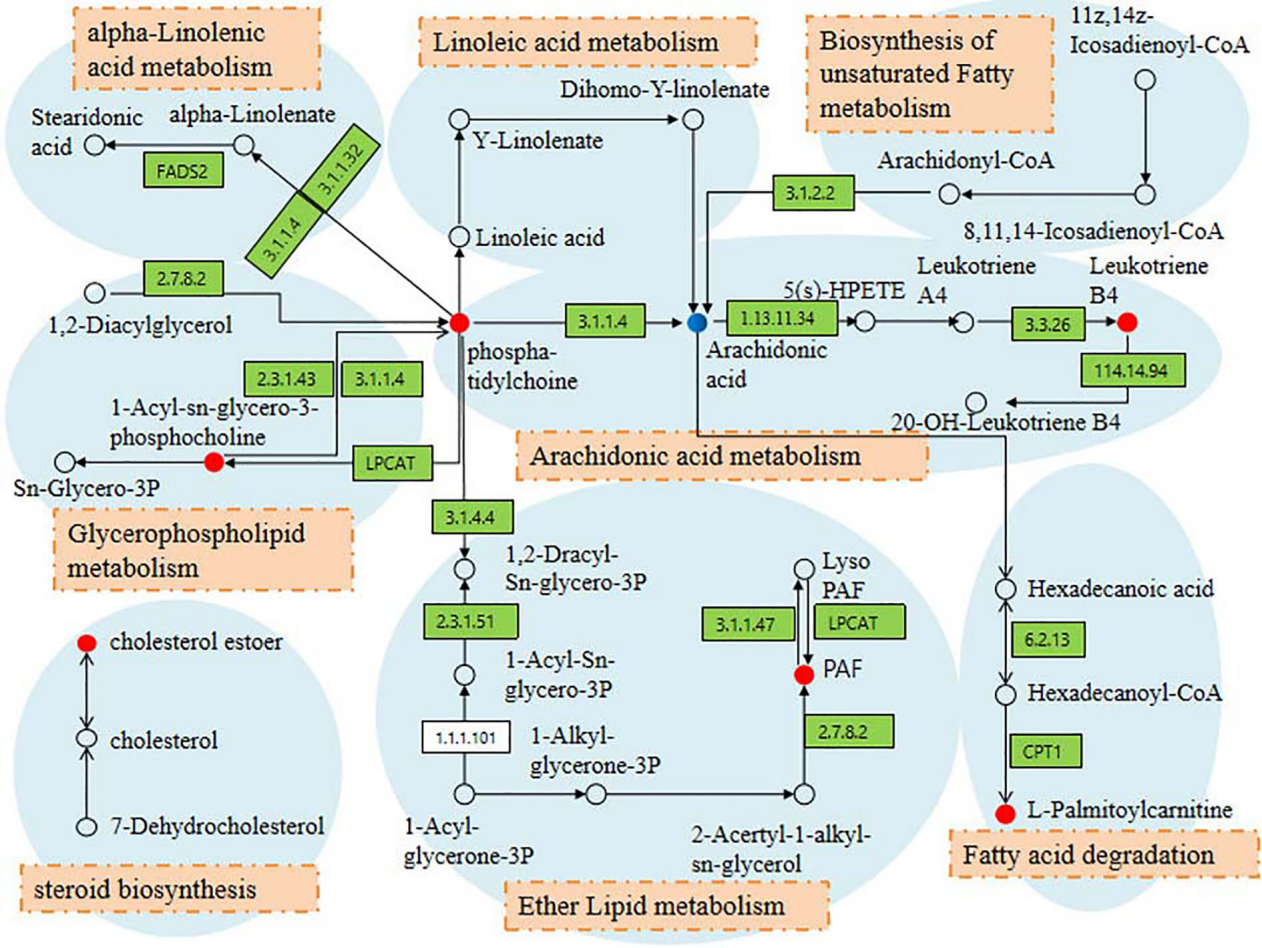
Correlation networks of the potential biomarkers and the relevant metabolic pathways. Red and blue dots indicate potential biomarker organisms found in this study. Red: the content in the model group is higher than the control group; blue: the content in the model group is lower than the control group. Orange filled boxes indicate related metabolic pathways.

**Figure 6:**
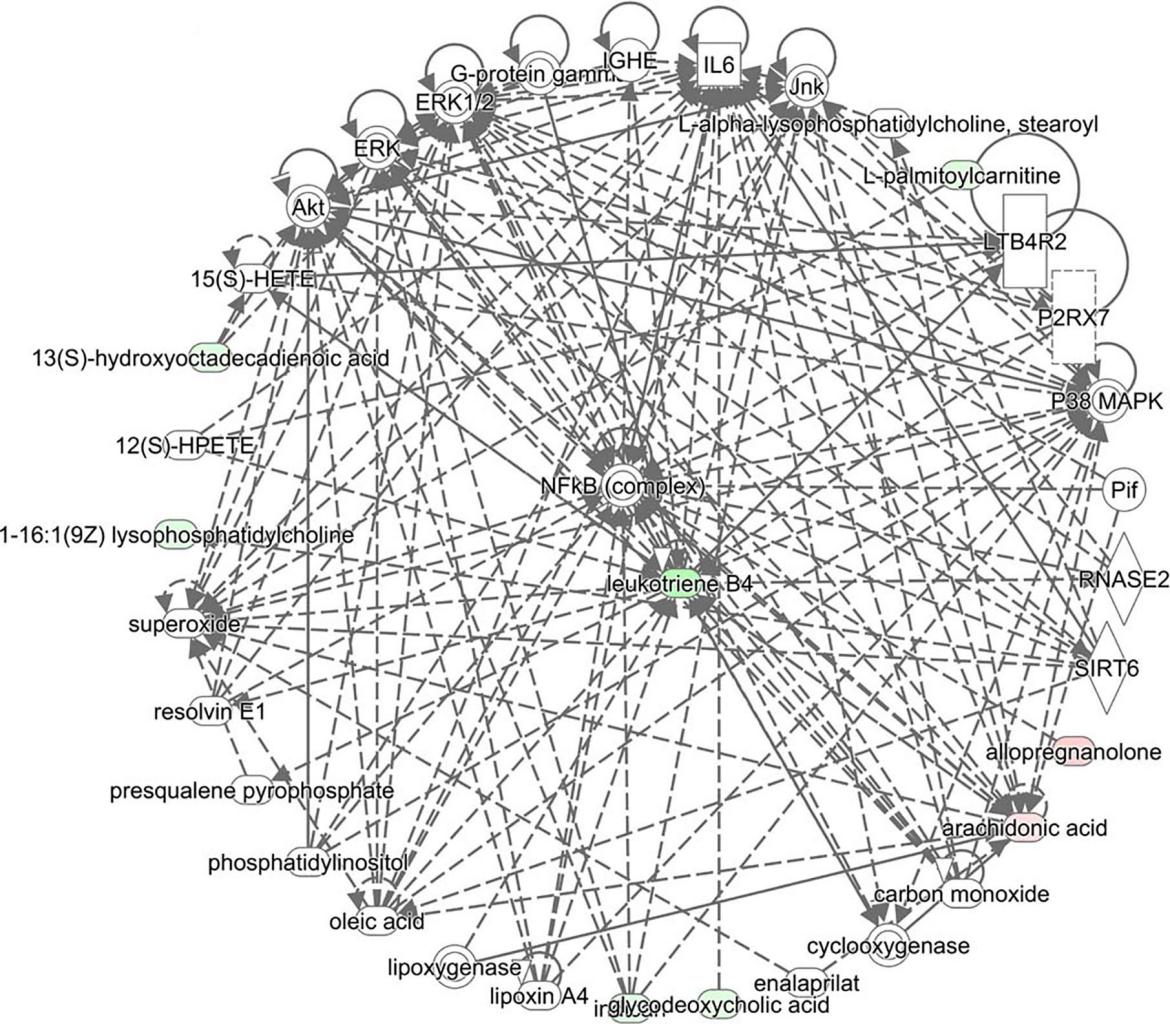
Biomarker metabolites network based on intelligent pathway analysis use IPA analysis. Light green represents low expression in diseases, light pink represents high expression in diseases, and the darker the color, the greater the degree.

Based on the 46 potential biomarkers identified, we used MetaboAnalyst to characterize the metabolic pathways associated with them as showed in **Figure 4**, a total of 8 metabolic pathways including linoleic acid metabolism, arachidonic acid metabolism, glycerol phospholipid metabolism, alpha-linolenic acid metabolism, ether lipid metabolism, steroid biosynthesis, fatty acid metabolism, biosynthesis of unsaturated fatty acids were characterized. The results revealed that changes in these metabolic pathways may be most relevant to metabolic changes throughout the pathology of OP. Among the marachidonic acid metabolism, glycerol phospholipid metabolism, and ether lipid metabolism were the metabolic pathways extremely related to the pathological process of OP. A metabolic network involving potential biomarkers involved in the pathogenesis of OP was established and summarized in this study (**Figure 5**). At the same time, these data of potential biomarkers with multiple changes were imported into the IPA software to construct an OP-related metabolic network in **Figure 6**. The results showed that the level of leukotriene B4 was down-regulated, at the same time, the level of allopregnanolone, arachidonic acid were up-regulated. As can be seen from the right figure, the occurrence of OP is closely related to leukotriene B4 and NF-κB protein.

### Effect of *Ext-Epi* on Metabolic Spectrum of OP

In order to evaluate whether Ext-epi can affect the metabolic pathway changes caused by OP and the degree of action of different doses of Ext-epi, PCA score charts of metabolic spectra of control group, model group, ZL-IH,ZL-IM,ZL-IL group are shown in **Figure S6**. From the score chart of PCA, it can be found that the control group and the model group have obvious separation trend, indicating that the serum metabolism spectrum of OP rat model after ovariectomy has obvious changes. However, clustering within the control group is not obvious. Positions of ZL-IH, ZL-IM, and ZL-IL groups are approach to the control group, which show that Ext-epi has certain effect on the pathological process of model rats with metabolic level after preventing and treating OP model rats. Among them, the ZL-IL group has obvious clustering and is closer to the control group. Although ZL-IH and ZL-IM have a callback trend to some extent, they are scattered and widely distributed in the group. In the 3D-PCA score chart (**Figure S7**), the spatial distribution of each group can be observed more clearly. In addition, different doses of Ext-epi have significant effects on the expression of potential biomarkers in OP model, making it callback to the control group, See **Table S3** for the changes of each biomarkers, and the direct view chart is shown in **Figure S8**. The results showed that the ZL-IH intervention could be recalled 38, among them 23 with statistical difference (p<0.05), the ZL-IM intervention could be recalled 43, among them 30 with statistical difference (p<0.05), the ZL-IL intervention could be adjusted 38, among them 26 with statistical difference (p<0.05). Three different doses of Ext-epi can reverse 37 potential biomarkers to different degrees and 16 have significant differences. It showed that different doses of therapeutic drugs can play a role in treating the progress of OP disease by interfering with the corresponding biomarkers. The main metabolic pathways for the common callback markers to participate in regulation arearachidonic acid metabolism, glycerolphospholipid metabolism, ether lipid metabolism, and these results suggest that the above metabolic pathway may be the target pathway for Ext-epi to protect OP.

## Discussion

OP is a disease that seriously affects the quality of life of the elderly (especially postmenopausal women). Its gradual increase in age increases the prevalence and even fractures. Therefore, searching for effective biomarkers and therapeutic drug interactions is very important. However there is no more effective detection method, BMD is still one of the commonly used indicators for monitoring and diagnosis of OP. This study found that Ext-epi can significantly improve the phenomenon of elevated levels of BMD, BMC, serum osteocalcin, and tumor necrosis factor-αlevels in OP rats, and can also significantly reduce the level of estradiol in the body, indicating that the occurrence of osteoporosis in OP rats may be closely related to the decrease of estradiol content in the body. It is speculated that Ext-epi may directly or indirectly regulate human bone metabolism and cytokines, thereby increasing BMD and BMC in OP rats. However, when estrogen is lacking in OP rats, the therapeutic effect of Ext-epi may be accompanied by a significant estrogen-like effect. This study analyzed rat serum based on UPLC-Q-TOF/MS metabolomics, and finally locked 46 potential biomarkers, 28 in ESI+ and 18 in ESI-. Pathways involved in metabolism include linoleic acid metabolism, arachidonic acid metabolism, glycerol phospholipid metabolism, alpha-linolenic acid metabolism, ether lipid metabolism, steroid biosynthesis, fatty acid metabolism, and biosynthesis of unsaturated fatty acids. After treatment with different doses of Ext-epi solution, the three groups can exchange 37 potential biomarkers. The main metabolic pathways involved include arachidonic acid metabolism, glycerol metabolism, ether lipid metabolism, and biosynthesis of unsaturated fatty acids.

Unsaturated fatty acids can be divided into two types: monounsaturated and polyunsaturated, which are essential fatty acids in the body. Arachidonic acid (AA), linolenic acid, and linoleic acid involved in this study all belong to polyunsaturated fatty acids, wherein linoleic acid can be converted into AA *in vivo*. Studies have shown that n6 series polyunsaturated fatty acids may enhance the growth of osteoblasts and increase bone density to reduce the risk of fracture (Liang et al., 2016c; Liang et al., 2016d). This study found that the content of polyunsaturated fatty acid metabolites in rats decreased significantly after ovariectomy, suggesting that the occurrence of OP may be closely related to unsaturated fatty acid metabolic disorders. AA is an essential unsaturated fatty acid with a high content in the cell membrane which can be metabolized to prostaglandins, thromboxane and leukotrienes through the cyclooxygenase pathway and lipoxygenase pathway (Zhang et al., 2012a). The metabolite produced by a specific enzyme catalyzes many important physiological processes, and plays a significant regulatory effect in inflammation and immune response. AA is produced by the activation of phospholipases under the action of inflammatory stimuli and inflammatory mediators such as C5a in inflammation, lysosomal neutrophils are an important source of phospholipase. AA is metabolized by the cyclooxygenase and lipid oxygenase pathways to produce various products. Studies have shown that AA may increase serum content after menopause and enhance its function of regulating inflammatory response, metabolic syndrome, blood pressure, bone remodeling, and platelet activation (Zhang et al., 2006). This study found that AA and its metabolites have undergone significant changes in the AA metabolic pathway. It is suggested that the AA metabolic pathway may be closely related to the metabolic changes of bone crisp.

Glycerol phospholipid is the phospholipid with the highest content in human body. It is not only an important component of biofilm, but also one of the bile and membrane surfactants involved in signal conduction and cell membrane recognition (Liang et al., 2017b). Glycerol phospholipids are synthesized mainly by two routes: (1) synthesis of cephalin and lecithin by the diglyceride synthesis pathway, and (2) synthesis of other glycerolphospholipids by the GDP-diglyceride synthesis pathway. Studies have shown that abnormal metabolism of glycerolphospholipids may be related to the metabolic mechanism of the risk of OP associated with primary age in mice (Jiang et al., 2020). More studies have shown that low bone density menopausal women and healthy human plasma lipids and polar metabolite profiles compared to low bone density menopausal women plasma levels of various glycerolphospholipid metabolites have changed, glycerolphospholipid metabolism possible is a potential mechanism for the reduction of bone mineral density in women with menopause. In addition, estradiol is a commonly used extract for the clinical relief of OP, which partially inhibits the activity of osteoclasts and its key role in bone resorption. Studies of the effects of estradiol on osteoclast metabolism have revealed that glycerolphospholipid metabolism is a major potential targeting pathway for estradiol, which is caused by changes in LCAT activity and estradiol. Lipid peroxidation products change to affect the metabolism of osteoclasts. In this study, the levels of estradiol and glycerophospholipid metabolites in OP rats were significantly decreased, while the content of Ext-epi extract increased after administration, indicating that the therapeutic effect of Ext-epi extract on osteoporosis may be to further regulate glycerophospholipid metabolism through estradiol hormone-like effect.

In the cardiovascular system, PAF has been proved to play an essential function in the occurrence and progress of platelet and neutrophil aggregation, vascular permeability, micro vascular leakage, thrombosis, and atherosclerosis. Ether grease is a unique type of glycerophospholipid, which has an alkyl chain and is connected to sn-1 *via* an ether bond. The alcohol group part connected to phosphate group in ether grease is usually choline or ethanolamine, and inositol or serine is occasionally observed (Thai et al., 1997). Ether lipids take in multifarious biological functions, including regulating cell differentiation, affecting cell signal transduction, and reducing oxidative stress through its potential endogenous antioxidant function (Liang et al., 2016b). In addition, it also plays an essential part in the tissue and stability of lipid rafts. Studies have shown that the ether lipid content in human and animal tumor cells is higher than that in healthy cells, which indicates that ether lipids may be involved in the pathogenesis of cancer (Benjamin et al., 2013). But the relationship between ether lipids and OP is rarely reported. This study found that the occurrence of OP may be related to ether lipid metabolic disorder, and Ext-epi can regulate this metabolic disorder and make the content of PAF tend to be normal to achieve the effect of treating OP.

IPA analysis results show that leukotriene B4 may be the core biomarker. Leukotriene B4 is one of the metabolic pathway products of arachidonic acid lipoxygenase, which can stimulate the production of pro-inflammatory cytokines and mediators and has the ability to enhance and prolong tissue inflammation (Crooks and Stockley, 1998). At the same time, leukotriene B4 is also one of the natural active ligands of peroxisome proliferator-activated receptor 2 (PPARΥ2) *in vivo*. It may promote the expression of osteoclast marker gene in bone marrow cells by activating the transcription activity of PPARΥ2, thus participating in the pathogenesis of OP (Chao and Xiaoming, 2018). In this experiment, the content of leukotriene B4 in OP model rats is significantly increased, which indicates that it may have inflammatory reaction and activate PPARΥ2 transcription activity, thus aggravating the degree of osteoporosis. However, the content of leukotriene B4 can be significantly reduced after treatment with Ext-epi, thus delaying the disease process. This marker may be a potential target for Ext-epi to treat OP, but it still needs further verification.

Metabolomics is a newly developed subject after genomics and proteomics and is an important part of systems biology (Zhang et al., 2012b; Zhang et al., 2016; Li et al., 2018). It is a powerful approach to explore the metabolic biomarkers and targets of natural products (Liang et al., 2016b; Liang et al., 2016e; Wang et al., 2016). In this study, UPLC- Q-TOF/MS was used to analyze the rats in the OP model and the rats treated with different doses of Ext-epi solution for better understand the influence of Ext-epion bone mass. The effect of metabolic changes during the development of crisp disease found that the poor metabolites in 46 were closely related to the OP syndrome, and the different metabolites in the different dose groups could significantly recall 16 different metabolites, among them, the treatment effect of ZL-IL group is relatively stable and the clustering among groups is relatively obvious, this dose can be considered as the better dose for further study. The preventive and therapeutic effects of Ext-epi on OP were evaluated from the metabolomics level.

## Conclusion

In this study, we used the method of ovariectomy to replicate the OP model. Based on UPLC-Q-TOF/MS and multivariate data analysis methods, 46 potential organisms were revealed from the rat serum metabolomics and marker 8 related metabolic pathways. Ext-epi in different dose groups have significant reversal effects on 16 potential biomarkers, involving three major metabolic pathways. Thereby affecting the metabolic level of OP rats, so that it develops in the direction of relieving OP. This study provides a basis for the development of Ext-epi for the treatment of OP, and also provides a reference for the development of extracts for the treatment of OP.

## Data Availability Statement

The raw data supporting the conclusions of this article will be made available by the authors, without undue reservation, to any qualified researcher.

## Ethics Statement

The animal study was reviewed and approved by Animal Care and Ethics Committee at Nanchang University.

## Author Contributions

G-CS conceived and designed the experiments. J-FZ, J-YX, Y-EX, S-LC, Y-XG, and Q-YG performed the experiment. J-FZ and J-YX analyzed the data. G-CS guided the experiment. J-FZ wrote the paper. All authors contributed to the article and approved the submitted version.

## Funding

This work was funded by the National Natural Science Foundation of China (No. 81960881; 81660801).

## Conflict of Interest

The authors declare that the research was conducted in the absence of any commercial or financial relationships that could be construed as a potential conflict of interest.
