# Supplementary material for: High-Throughput Metabolomics Method for Discovering Metabolic Biomarkers and Pathways to Reveal Effects and Molecular Mechanism of Ethanol Extract From Epimedium Against Osteoporosis
Source: Front Pharmacol. 2020 Aug 27;11:1318. doi: 10.3389/fphar.2020.01318 (PMC7481463; doi:10.3389/fphar.2020.01318)
Supplement: Supplementary file 1 [file Table_1.docx]

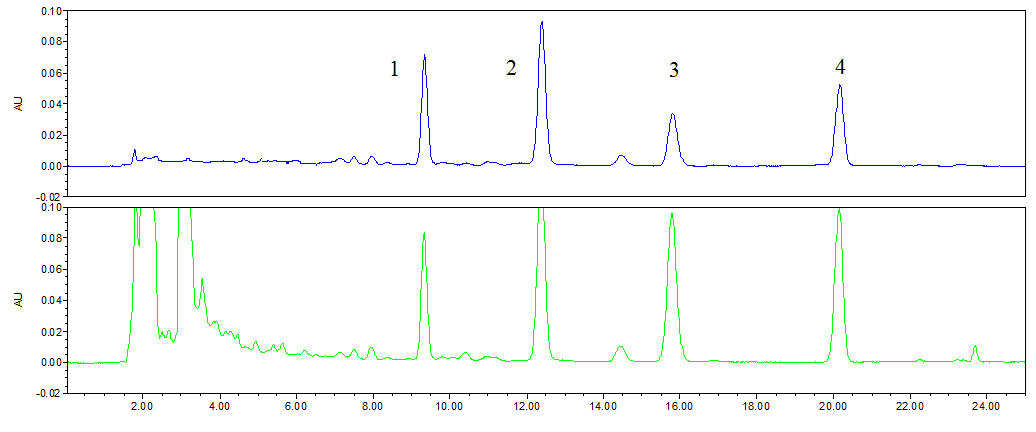


**Fig. S1** HPLC chromatograms of four active components (up) and sample (down).

Note: 1, epimedin A; 2, epimedin B; 3, epimedin C; 4, icariin.

Chromatographic conditions: Agilent C18 column (4.6 mm × 250 mm, 5 μm); mobile phase acetonitrile (A)-0.1% phosphoric acid solution (B), gradient elution (0-15 min, 40 % A; 16 -24 min, 40%-50% A ); flow rate 1.0 mL/min; measuring wavelength 270 nm, column temperature 35℃; injection volume 10 uL.


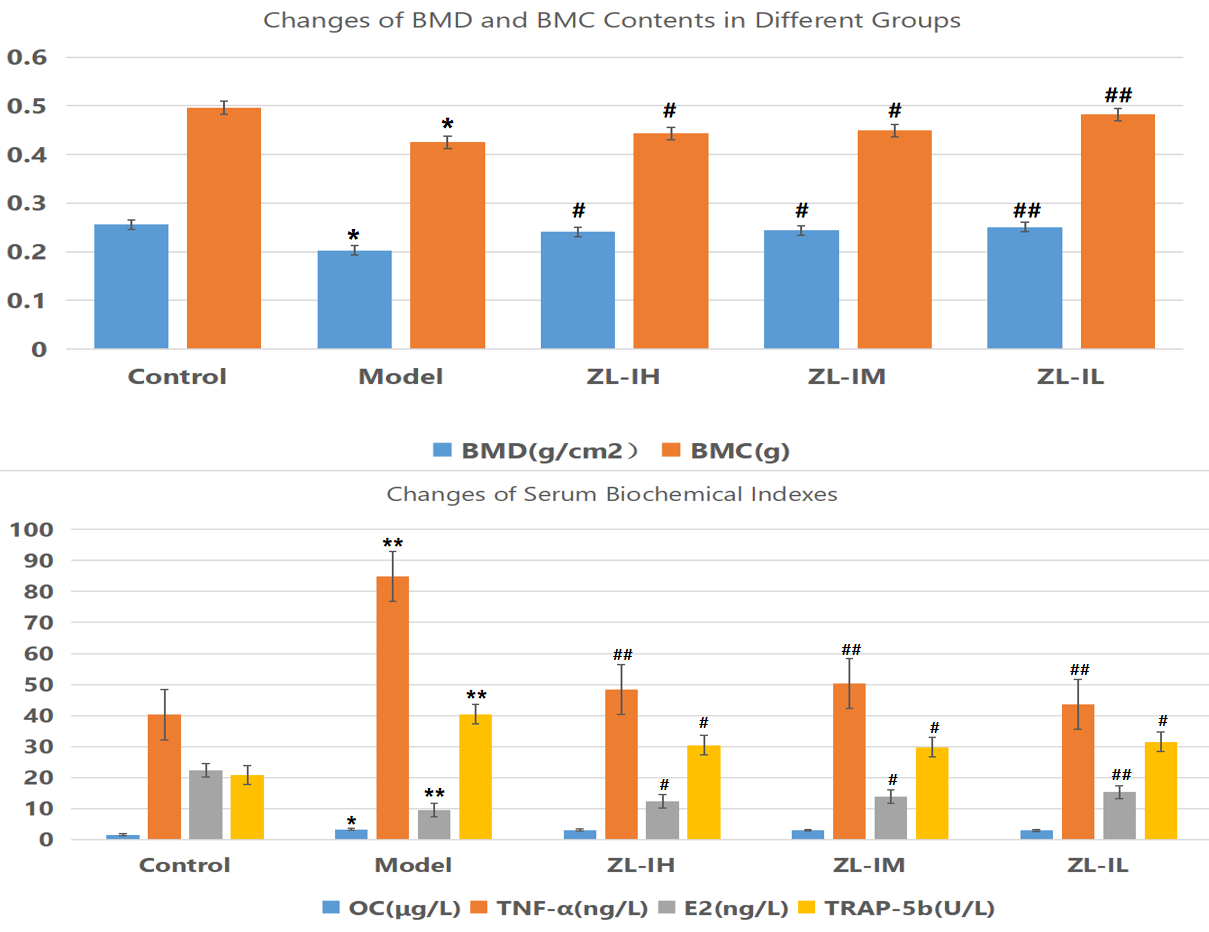


**Fig.S2** Effects of *Epimedium* extracts on BMD, BMC and bone metabolism index in ovariectomized osteoporosis rats.

*: vs control group, the content of model group has significant difference, p<0.05.

**: vs control group, the content of model group has very significant difference, p<0.01.

#: vs model group, the content in the treatment group is significantly different, p<0.05.

##: vs model group, the content in the treatment group is very significantly different, p<0.01.


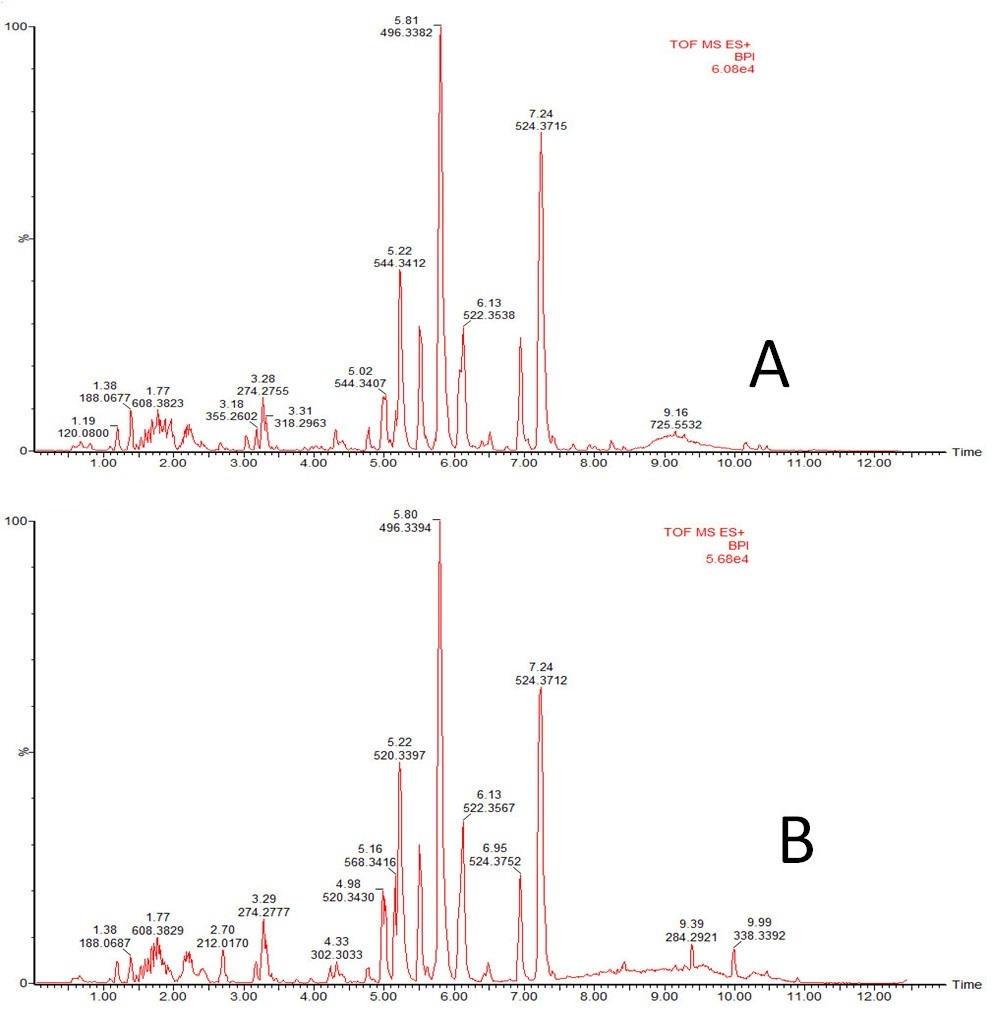


**Fig.S3** Serum UPLC-MS BPI ion map in positive ion mode. (A)Control group rat serum. (B) Model group rat serum.


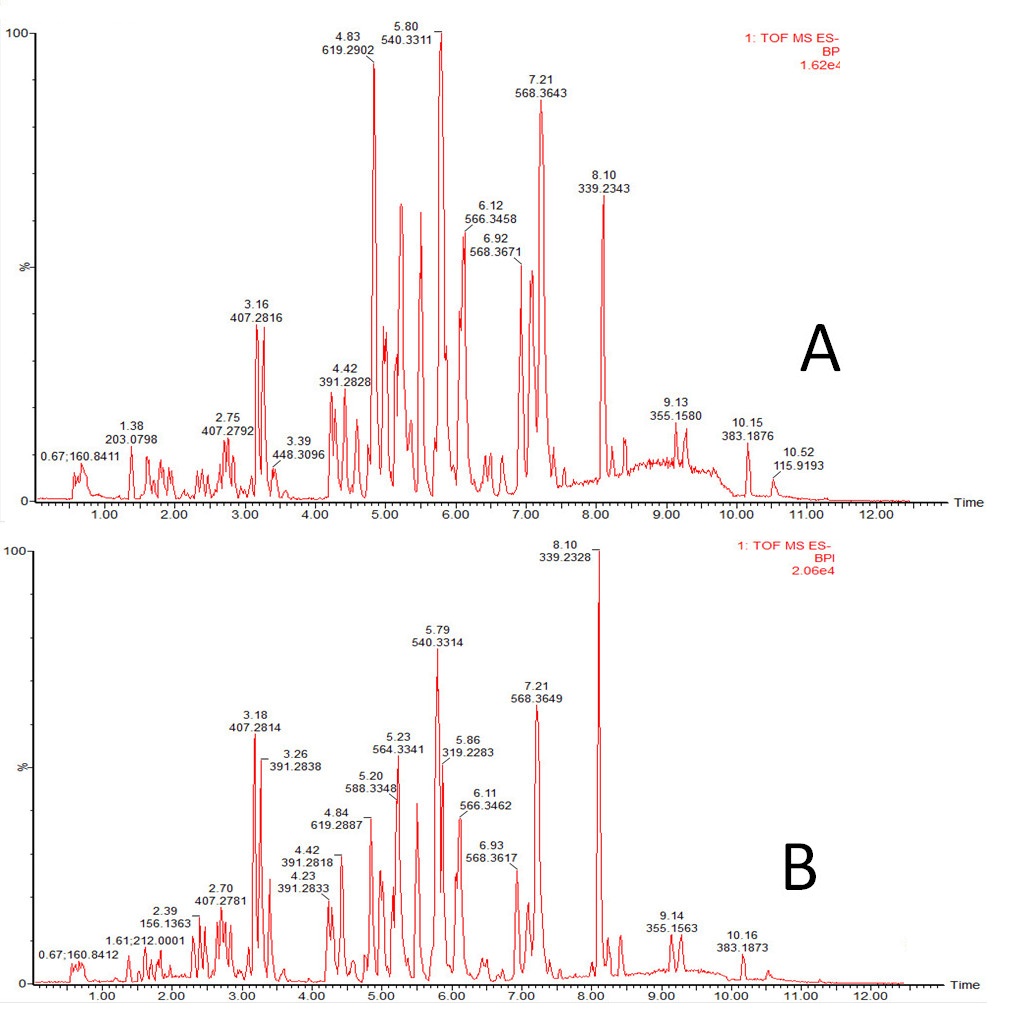


**Fig.S4.** Serum UPLC-MS BPI ion map in negative ion mode. (A)Control group rat serum. (B) Model group rat serum.


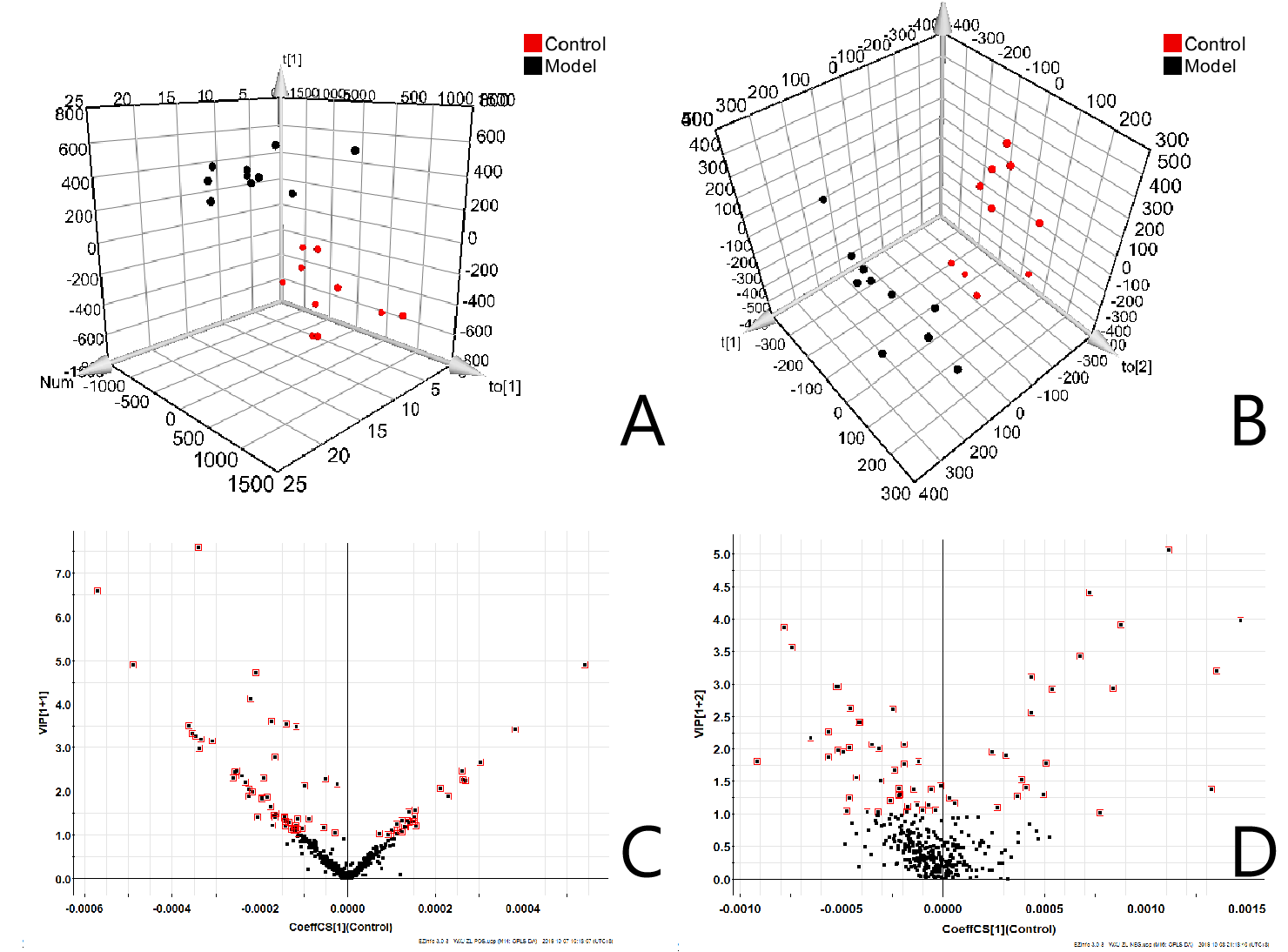


**Fig.S5.** 3D-Score plot by the PCA analyse and VIP plot by OPLS-DA analyse between the control group and model group. (A)3D-Score plot in positive ion mode (B)3D-Score plot in negative ion mode. (C) VIP plot in positive ion mode. (D) VIP plot in negative ion mode


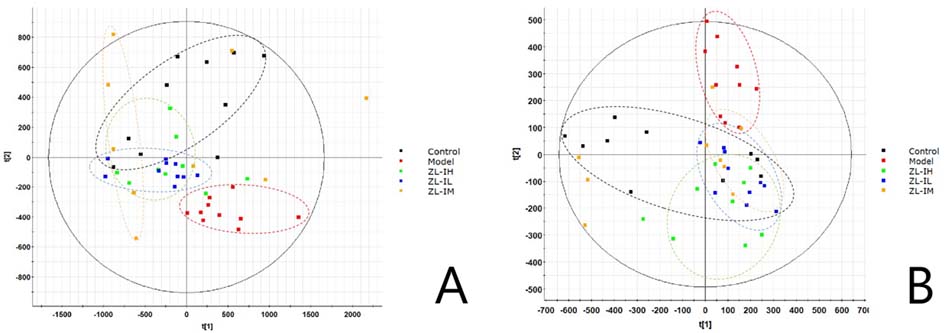


**Fig.S6.** PCA scores plot of serum metabolism profile of rats with control group, model group and different doses of Epimedium in both positive and negative mode. (A) Positive ion mode. (B) Negative ion mode.


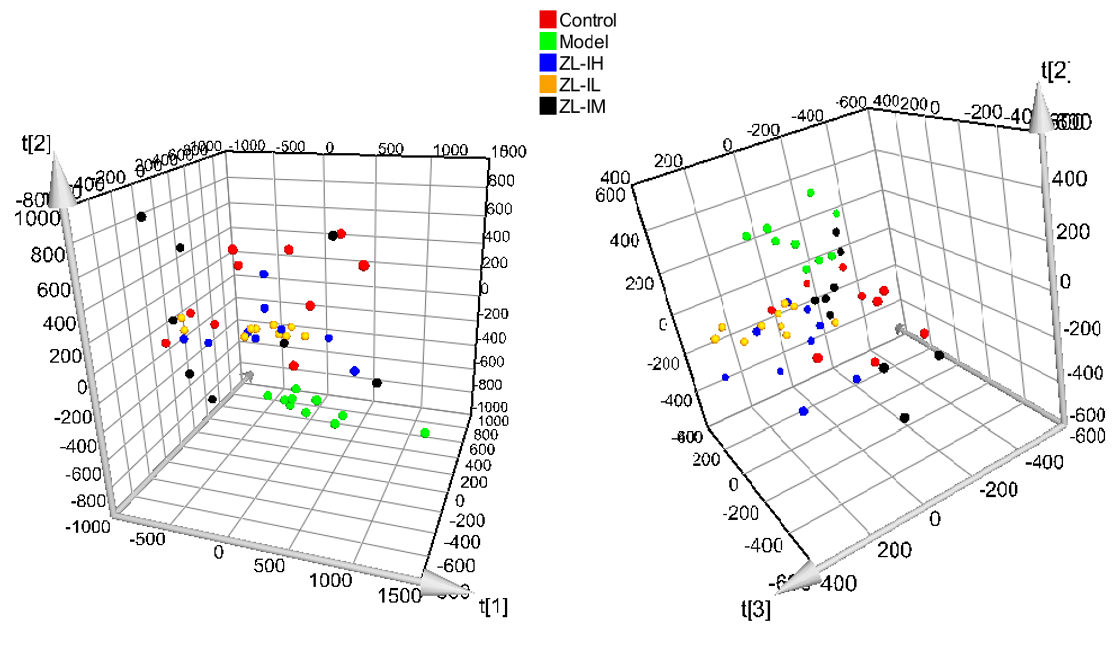


**Fig.S7.** 3D-Score plot by the PCA analyse of all group. (A)3D-Score plot in positive ion mode (B)3D-Score plot in negative ion mode.


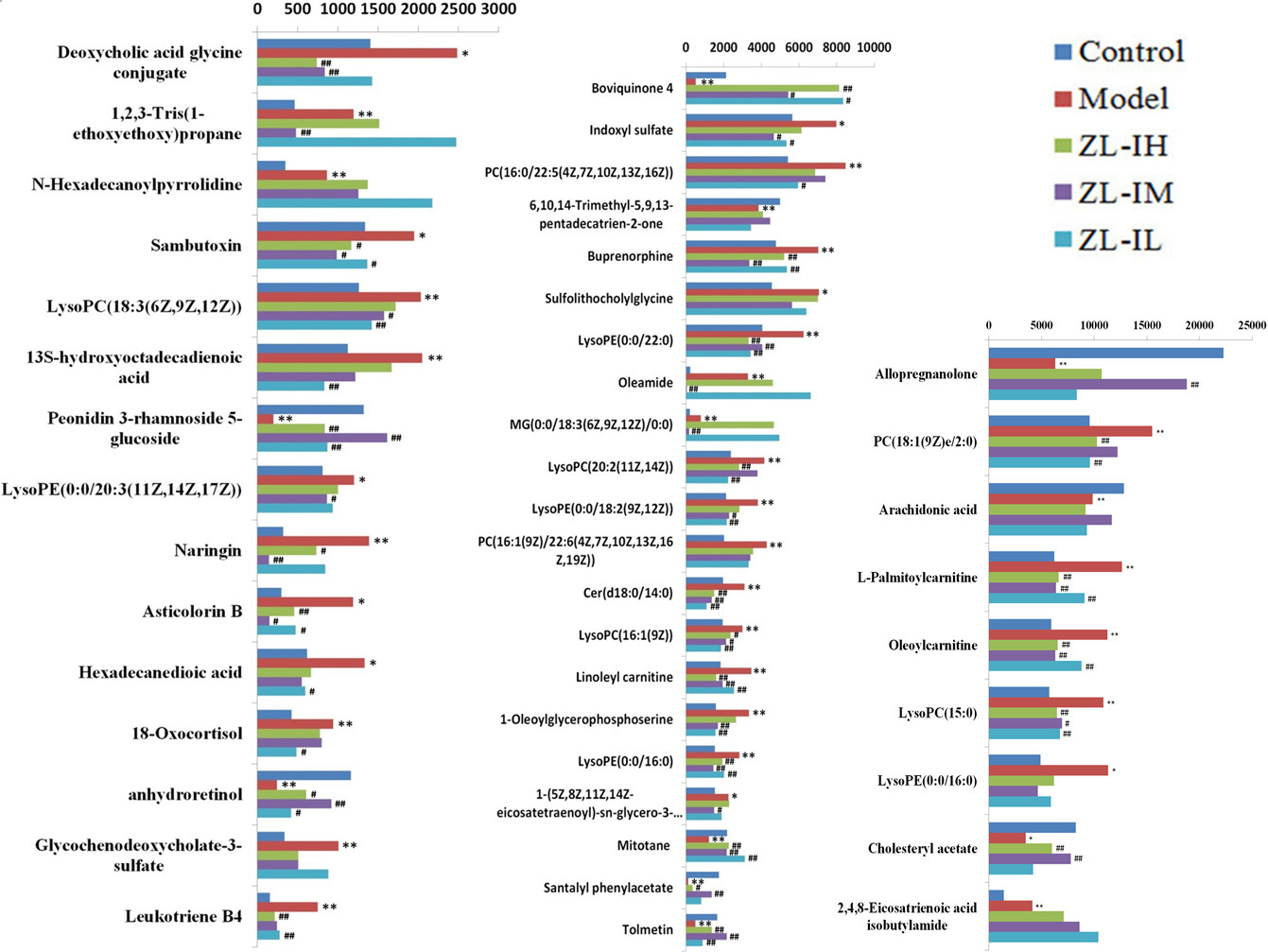


**Fig.S8**. Changes in serum content of potential biomarkers after oral different doses of Epimedium. (Compare with control group，*P<0.05，**P<0.01；Compare with model group,# P<0.05，##P<0.01）

Table S1: Effects of *Epimedium* extracts on BMD, BMC and bone metabolism index in ovariectomized osteoporosis rats

| Group | Number | Bonemineral density（BMD）  (g/cm^2^) | Bne mineral content (BMC)  (g) | Osteocalcin  (Mg/L) | Tumornecrosis  Factord-α  (ng/L) | Estrogen  (ng/L) | TRAP  (U/L) |
| --- | --- | --- | --- | --- | --- | --- | --- |
| Control | 10 | 0.256±0.031 | 0.496±0.035 | 1.47±0.47 | 40.23±11.76 | 22.34±7.16 | 20.764±7.431 |
| Model | 10 | 0.203±0.022^*^ | 0.425±0.024^*^ | 3.21±0.54^*^ | 84.9±20.37^**^ | 9.45±1.23^**^ | 432±10.731^**^ |
| Zl-IH | 9 | 0.241±0.013^#^ | 0.443±0.017^#^ | 2.98±0.32 | 48.45±20.44^##^ | 12.31±0.89^#^ | 30.417±8.426^#^ |
| Zl-IM | 9 | 0.244±0.031^#^ | 0.449±0.034^#^ | 2.96±0.24 | 50.38±18.49^##^ | 13.76±1.23^#^ | 29.746±6.294^#^ |
| Zl-IL | 10 | 0.251±0.017^##^ | 0.482±0.019^##^ | 2.88±0.36^#^ | 43.59±13.24^##^ | 15.24±1.22^#^ | 31.465±9.287^#^ |

*: Compared with the control group, the content of model group has significant difference,p<0.05.

**: Compared with the control group, the content of model group has very significant difference,p<0.01.

**#:** Compared with the model group, the content in the treatment group is significantly different,p<0.05.

**##:** Compared with the model group, the content in the treatment group is very significantly different,p<0.01

Table S2：Potential biomarker information of osteoporosis using ultra-high performance liquid chromatography combined with quadrupole time-of-flight mass spectrometry.

|  | Rt/min | Detection m/z | Practical  m/z | Scan mode | M+X | Mass Error (ppm) | Proposed composition | Predicting compound | HMDB ID | T-Test/p  valve | VIP | Chang trend |
| --- | --- | --- | --- | --- | --- | --- | --- | --- | --- | --- | --- | --- |
| 1 | 0.66 | 258.1127 | 257.2845 | ESI+ | M+H | -2.31 | C_15_H_15_NO_3_ | Tolmetin | HMDB0014643 | 2.56×10^-3^ | 1.17 | ↓ |
| 2 | 3.64 | 339.2318 | 338.4831 | ESI+ | M+H | 4.15 | C_23_H_30_O_2_ | Santalylphenylacetate | HMDB0032502 | 4.06×10^-6^ | 1.64 | ↓ |
| 3 | 4.41 | 468.3106 | 467.6401 | ESI+ | M+H | 5.21 | C_29_H_41_NO_4_ | Buprenorphine | HMDB0015057 | 6.59×10^-3^ | 1.78 | ↑ |
| 4 | 4.53 | 494.3255 | 493.6142 | ESI+ | M+H | 2.01 | C_24_H_48_NO_7_P | LysoPC(16:1(9Z)) | HMDB0010383 | 3.37×10^-3^ | 1.24 | ↑ |
| 5 | 4.63 | 518.3250 | 517.6356 | ESI+ | M+H | 5.23 | C_26_H_48_NO_7_P | LysoPC(18:3(6Z,9Z,12Z)) | HMDB0010387 | 1.28×10^-3^ | 1.09 | ↑ |
| 6 | 5.10 | 481.3192 | 481.6035 | ESI+ | M+H | 3.24 | C_23_H_48_NO_7_P | LysoPC(15:0) | HMDB0010381 | 1.27×10^-3^ | 2.53 | ↑ |
| 7 | 5.65 | 424.3436 | 423.6291 | ESI+ | M+H | 6.13 | C_25_H_45_NO_4_ | Linoleyl carnitine | HMDB0006469 | 3.3×10^-4^ | 1.60 | ↑ |
| 8 | 5.75 | 453.2871 | 453.5503 | ESI+ | M+H | -4.12 | C_21_H_44_NO_7_P | LysoPE(0:0/16:0) | HMDB0011473 | 6.24×10^-5^ | 1.38 | ↑ |
| 9 | 6.18 | 400.3428 | 399.6077 | ESI+ | M+H | -2.14 | C_23_H_45_NO_4_ | L-Palmitoylcarnitine | HMDB0000222 | 1.35×10^-3^ | 2.83 | ↑ |
| 10 | 6.41 | 426.3594 | 425.654 | ESI+ | M+H | 4.52 | C_25_H_47_NO_4_ | Oleoylcarnitine | HMDB0005065 | 7.42×10_-4_ | 2.73 | ↑ |
| 11 | 6.48 | 548.3724 | 547.7046 | ESI+ | M+H | 5.98 | C_28_H_54_NO_7_P | LysoPC(20:2(11Z,14Z)) | HMDB0010392 | 9.57×10^-4^ | 1.50 | ↑ |
| 12 | 7.43 | 549.3804 | 549.7205 | ESI+ | M+H | 6.21 | C_28_H_56_NO_7_P | PC(18:1(9Z)e/2:0) | HMDB0011148 | 3.31×0^-3^ | 2.77 | ↑ |
| 13 | 7.62 | 282.2798 | 281.4766 | ESI+ | M+H | 4.89 | C_18_H_35_NO | Oleamide | HMDB0002117 | 3.61×10^-8^ | 2.34 | ↑ |
| 14 | 7.94 | 537.3807 | 537.7098 | ESI+ | M+H | 4.36 | C_27_H_56_NO_7_P | LysoPE(0:0/22:0) | HMDB0011490 | 2.76×10^-3^ | 1.80 | ↑ |
| 15 | 8.11 | 804.5538 | 804.0872 | ESI+ | M+H | -3.48 | C_46_H_78_NO_8_P | PC(16:1(9Z)/22:6(4Z,7Z,10Z,13Z,16Z,19Z)) | HMDB0008023 | 4.53×10^-3^ | 1.57 | ↑ |
| 16 | 8.13 | 309.2264 | 308.4110 | ESI+ | M+H | 6.34 | C_15_H_32_O_6_ | 1,2,3-Tris(1-ethoxyethoxy)propane | HMDB0037162 | 4.6×10^-4^ | 1.01 | ↑ |
| 17 | 8.22 | 353.2691 | 352.5081 | ESI+ | M+H | 7.21 | C_21_H_36_O_4_ | MG(0:0/18:3(6Z,9Z,12Z)/0:0) | HMDB0011539 | 5.9×10^-5^ | 1.01 | ↑ |
| 18 | 8.25 | 305.2473 | 304.4669 | ESI+ | M+H | 2.31 | C_20_H_32_O_2_ | Arachidonic acid | HMDB0001043 | 1.53×10^-3^ | 1.92 | ↓ |
| 19 | 8.43 | 263.2376 | 262.4302 | ESI+ | M+H | 3.45 | C_18_H_30_O | 6,10,14-Trimethyl-5,9,13-pentadecatrien-2-one | HMDB0034495 | 4.64×10^-3^ | 1.13 | ↓ |
| 20 | 8.90 | 310.3111 | 309.5298 | ESI+ | M+H | 1.61 | C_20_H_39_NO | N-Hexadecanoylpyrrolidine | HMDB0032740 | 3.73×10^-7^ | 1.19 | ↑ |
| 21 | 9.14 | 512.5021 | 511.8634 | ESI+ | M+H | 2.78 | C_32_H_65_NO_3_ | Cer(d18:0/14:0) | HMDB0011759 | 1.21×10^-3^ | 1.20 | ↑ |
| 22 | 9.37 | 362.3429 | 361.6043 | ESI+ | M+H | 3.45 | C_24_H_43_NO | 2,4,8-Eicosatrienoic acid isobutylamide | HMDB0030385 | 1.23×10^-6^ | 2.52 | ↑ |
| 23 | 9.65 | 319.2637 | 318.4935 | ESI+ | M+H | 2.47 | C_21_H_34_O_2_ | Allopregnanolone | HMDB0001449 | 2.09×10^-4^ | 4.72 | ↓ |
| 24 | 9.65 | 269.2276 | 268.4363 | ESI+ | M+H | 5.75 | C_20_H_28_ | anhydroretinol | HMDB0062447 | 1.09×10^-4^ | 1.14 | ↓ |
| 25 | 10.17 | 413.2666 | 412.5616 | ESI+ | M+H | -5.38 | C_26_H_36_O_4_ | Boviquinone 4 | HMDB0030057 | 2.01×10^-4^ | 1.49 | ↓ |
| 26 | 12.43 | 429.3730 | 428.6902 | ESI+ | M+H | 5.64 | C_29_H_48_O_2_ | Cholesteryl acetate | HMDB0003822 | 2.7×10^-10^ | 3.00 | ↓ |
| 27 | 12.87 | 610.1922 | 609.5526 | ESI+ | M+H | -4.12 | C_28_H_33_O_15_^+^ | Peonidin 3-rhamnoside 5-glucoside | HMDB0038090 | 3.13×10^-5^ | 1.30 | ↓ |
| 28 | 12.89 | 808.5850 | 808.1189 | ESI+ | M+H | -6.24 | C_46_H_82_NO_8_P | PC(16:0/22:5(4Z,7Z,10Z,13Z,16Z)) | HMDB0007989 | 3.39×10^-3^ | 1.88 | ↑ |
| 29 | 0.56 | 316.9477 | 320.0410 | ESI- | M-H | 8.21 | C_14_H_10_C_l4_ | Mitotane | HMDB0014786 | 9.27×10^-5^ | 1.74 | ↓ |
| 30 | 0.68 | 142.9902 | 144.5560 | ESI- | M-H | 2.63 | C_6_H_5_ClO_2_ | 4-Chlorocatechol | HMDB0041810 | 1.87×10^-2^ | 1.05 | ↓ |
| 31 | 1.61 | 212.0016 | 213.2100 | ESI- | M-H | 1.97 | C_8_H_7_NO_4_S | Indoxyl sulfate | HMDB0000682 | 2.37×10^-2^ | 2.17 | ↑ |
| 32 | 1.70 | 535.1780 | 536.5712 | ESI- | M-H | 4.36 | C_33_H_28_O_7_ | Asticolorin B | HMDB0030134 | 1.58×10^-2^ | 1.23 | ↑ |
| 33 | 1.90 | 579.1727 | 580.5346 | ESI- | M-H | 5.21 | C_27_H_32_O_14_ | Naringin | HMDB0002927 | 5.6×10^-5^ | 2.08 | ↑ |
| 34 | 2.18 | 512.2698 | 513.6870 | ESI- | M-H | 3.54 | C_26_H_43_NO_7_S | Sulfolithocholylglycine | HMDB0002639 | 1.51×10^-2^ | 2.3 | ↑ |
| 35 | 2.57 | 448.3070 | 449.6233 | ESI- | M-H | 3.96 | C_26_H_43_NO_5_ | Deoxycholic acid glycine conjugate | HMDB0000631 | 3.84×10^-2^ | 1.47 | ↑ |
| 36 | 2.61 | 528.2633 | 529.6870 | ESI- | M-H | -2.89 | C_26_H_43_NO_8_S | Glycochenodeoxycholate-3-sulfate | HMDB0002497 | 4.82×10^-3^ | 1.34 | ↑ |
| 37 | 2.72 | 375.1828 | 376.4434 | ESI- | M-H | -4.12 | C_21_H_28_O_6_ | 18-Oxocortisol | HMDB0000332 | 6.79×10^-3^ | 1.1 | ↑ |
| 38 | 3.14 | 335.2220 | 336.4657 | ESI- | M-H | 5.76 | C_20_H_32_O_4_ | Leukotriene B4 | HMDB0001085 | 6.02×10^-3^ | 1.15 | ↑ |
| 39 | 4.53 | 286.2149 | 286.4070 | ESI- | M-H | 2.64 | C_16_H_30_O_4_ | Hexadecanedioic acid | HMDB0000672 | 2.75×10^-2^ | 1.18 | ↑ |
| 40 | 4.62 | 502.2915 | 503.6090 | ESI- | M-H | -5.98 | C_25_H_46_NO_7_P | LysoPE(0:0/20:3(11Z,14Z,17Z)) | HMDB0011484 | 2.27×10^-2^ | 1.1 | ↑ |
| 41 | 4.98 | 520.2698 | 523.6040 | ESI- | M-H | 2.47 | C_24_H_44_NO_9_P_2_ | 1-Oleoylglycerophosphoserine | HMDB0061694 | 2.1×10^-3^ | 2.14 | ↑ |
| 42 | 5.00 | 457.2378 | 458.5320 | ESI- | M-H | 2.46 | C_23_H_39_O_7_P | 1-(5Z,8Z,11Z,14Z-eicosatetraenoyl)-sn-glycero-3-phosphate | HMDB0062312 | 4.69×10^-2^ | 1.22 | ↑ |
| 43 | 5.17 | 476.2798 | 477.5717 | ESI- | M-H | 3.58 | C_23_H_44_NO_7_P | LysoPE(0:0/18:2(9Z,12Z)) | HMDB0011477 | 2.51×10^-3^ | 2.18 | ↑ |
| 44 | 5.36 | 295.2285 | 296.4449 | ESI- | M-H | 6.48 | C_18_H_32_O_3_ | 13S-hydroxyoctadecadienoic acid | HMDB0004667 | 1.12×10^-2^ | 1.43 | ↑ |
| 45 | 5.45 | 452.2798 | 453.6136 | ESI- | M-H | 5.98 | C_28_H_39_NO_4_ | Sambutoxin | HMDB0041085 | 3.5×10^-2^ | 1.15 | ↑ |
| 46 | 5.74 | 452.2800 | 453.5503 | ESI- | M-H | 4.76 | C_21_H_44_NO_7_P | LysoPE(0:0/16:0) | HMDB0011473 | 3.8×10^-2^ | 3.44 | ↑ |

Note: ↑:The content of the model group was higher than that of the blank group.

↓:The content of the model group was lower than that of the blank group.

Table S3：Changes of metabolic markers in different dose groups of epimedium intervention.

|  | Rt/min | Detection m/z | Proposed composition | Predicting compound | HMDB ID | Trend | ZL-IH | ZL-IM | ZL-IL |
| --- | --- | --- | --- | --- | --- | --- | --- | --- | --- |
| 1 | 0.66 | 258.1127 | C_15_H_15_NO_3_ | Tolmetin | HMDB0014643 | ↓** | ## | ## | ## |
| 2 | 3.64 | 339.2318 | C_23_H_30_O_2_ | Santalylphenylacetate | HMDB0032502 | ↓** | # | ## | - |
| 3 | 4.41 | 468.3106 | C_29_H_41_NO_4_ | Buprenorphine | HMDB0015057 | ↑** | ## | ## | ## |
| 4 | 4.53 | 494.3255 | C_24_H_48_NO_7_P | LysoPC(16:1(9Z)) | HMDB0010383 | ↑** | # | # | ## |
| 5 | 4.63 | 518.325 | C_26_H_48_NO_7_P | LysoPC(18:3(6Z,9Z,12Z)) | HMDB0010387 | ↑** | - | # | ## |
| 6 | 5.1 | 481.3192 | C_23_H_48_NO_7_P | LysoPC(15:0) | HMDB0010381 | ↑** | ## | # | ## |
| 7 | 5.65 | 424.3436 | C_25_H_45_NO_4_ | Linoleyl carnitine | HMDB0006469 | ↑** | ## | ## | ## |
| 8 | 5.75 | 453.2871 | C_21_H_44_NO_7_P | LysoPE(0:0/16:0) | HMDB0011473 | ↑** | ## | ## | ## |
| 9 | 6.18 | 400.3428 | C_23_H_45_NO_4_ | L-Palmitoylcarnitine | HMDB0000222 | ↑** | ## | ## | ## |
| 10 | 6.41 | 426.3594 | C_25_H_47_NO_4_ | Oleoylcarnitine | HMDB0005065 | ↑** | ## | ## | ## |
| 11 | 6.48 | 548.3724 | C_28_H_54_NO_7_P | LysoPC(20:2(11Z,14Z)) | HMDB0010392 | ↑** | ## | - | ## |
| 12 | 7.43 | 549.3804 | C_28_H_56_NO_7_P | PC(18:1(9Z)e/2:0) | HMDB0011148 | ↑** | ## | - | ## |
| 13 | 7.62 | 282.2798 | C_18_H_35_NO | Oleamide | HMDB0002117 | ↑** | - | ## | # |
| 14 | 7.94 | 537.3807 | C_27_H_56_NO_7_P | LysoPE(0:0/22:0) | HMDB0011490 | ↑** | ## | ## | ## |
| 15 | 8.11 | 804.5538 | C_46_H_78_NO_8_P | PC(16:1(9Z)/22:6(4Z,7Z,10Z,13Z,16Z,19Z)) | HMDB0008023 | ↑** | - | - | - |
| 16 | 8.13 | 309.2264 | C_15_H_32_O_6_ | 1,2,3-Tris(1-ethoxyethoxy)propane | HMDB0037162 | ↑** | - | ## | # |
| 17 | 8.22 | 353.2691 | C_21_H_36_O_4_ | MG(0:0/18:3(6Z,9Z,12Z)/0:0) | HMDB0011539 | ↑** | ## | ## | ## |
| 18 | 8.25 | 305.2473 | C_20_H_32_O_2_ | Arachidonic acid | HMDB0001043 | ↓** | - | - | - |
| 19 | 8.43 | 263.2376 | C_18_H_30_O | 6,10,14-Trimethyl-5,9,13-pentadecatrien-2-one | HMDB0034495 | ↓** | - | - | - |
| 20 | 8.9 | 310.3111 | C_20_H_39_NO | N-Hexadecanoylpyrrolidine | HMDB0032740 | ↑** | - | - | ## |
| 21 | 9.14 | 512.5021 | C_32_H_65_NO_3_ | Cer(d18:0/14:0) | HMDB0011759 | ↑** | ## | ## | ## |
| 22 | 9.37 | 362.3429 | C_24_H_43_NO | 2,4,8-Eicosatrienoic acid isobutylamide | HMDB0030385 | ↑** | - | ## | ## |
| 23 | 9.65 | 319.2637 | C_21_H_34_O_2_ | Allopregnanolone | HMDB0001449 | ↓** | - | ## | - |
| 24 | 9.65 | 269.2276 | C_20_H_28_ | anhydroretinol | HMDB0062447 | ↓** | # | ## | # |
| 25 | 10.17 | 413.2666 | C_26_H_36_O_4_ | Boviquinone 4 | HMDB0030057 | ↓** | ## | # | # |
| 26 | 12.43 | 429.373 | C_29_H_48_O_2_ | Cholesteryl acetate | HMDB0003822 | ↓** | ## | ## | - |
| 27 | 12.87 | 610.1922 | C_28_H_33_O_15_^+^ | Peonidin3-rhamnoside 5-glucoside | HMDB0038090 | ↓** | ## | ## | ## |
| 28 | 12.89 | 808.585 | C_46_H_82_NO_8_P | PC(16:0/22:5(4Z,7Z,10Z,13Z,16Z)) | HMDB0007989 | ↑** | - | - | # |
| 29 | 0.56 | 316.9477 | C_14_H_10_C_l4_ | Mitotane | HMDB0014786 | ↓** | ## | ## | ## |
| 30 | 0.68 | 142.9902 | C_6_H_5_ClO_2_ | 4-Chlorocatechol | HMDB0041810 | ↓** | - | - | - |
| 31 | 1.61 | 212.0016 | C_8_H_7_NO_4_S | Indoxyl sulfate | HMDB0000682 | ↑** | - | # | # |
| 32 | 1.7 | 535.178 | C_33_H_28_O_7_ | Asticolorin B | HMDB0030134 | ↑** | ## | # | # |
| 33 | 1.9 | 579.1727 | C_27_H_32_O_14_ | Naringin | HMDB0002927 | ↑** | # | ## | - |
| 34 | 2.18 | 512.2698 | C_26_H_43_NO_7_S | Sulfolithocholylglycine | HMDB0002639 | ↑* | - | - | - |
| 35 | 2.57 | 448.307 | C_26_H_43_NO_5_ | Deoxycholic acid glycine conjugate | HMDB0000631 | ↑* | ## | # | - |
| 36 | 2.61 | 528.2633 | C_26_H_43_NO_8_S | Glycochenodeoxycholate-3-sulfate | HMDB0002497 | ↑** | - | - | - |
| 37 | 2.72 | 375.1828 | C_21_H_28_O_6_ | 18-Oxocortisol | HMDB0000332 | ↑** | - | - | # |
| 38 | 3.14 | 335.222 | C_20_H_32_O_4_ | Leukotriene B4 | HMDB0001085 | ↑** | ## | - | # |
| 39 | 4.53 | 286.2149 | C_16_H_30_O_4_ | Hexadecanedioic acid | HMDB0000672 | ↑* | - | - | # |
| 40 | 4.62 | 502.2915 | C_25_H_46_NO_7_P | LysoPE(0:0/20:3(11Z,14Z,17Z)) | HMDB0011484 | ↑* | - | # | - |
| 41 | 4.98 | 520.2698 | C_24_H_44_NO_9_P_2_ | 1-Oleoylglycerophosphoserine | HMDB0061694 | ↑** | - | ## | ## |
| 42 | 5 | 457.2378 | C_23_H_39_O_7_P | 1-(5Z,8Z,11Z,14Z-eicosatetraenoyl)-sn-glycero-3-phosphate | HMDB0062312 | ↑* | - | # | - |
| 43 | 5.17 | 476.2798 | C_23_H_44_NO_7_P | LysoPE(0:0/18:2(9Z,12Z)) | HMDB0011477 | ↑** | - | # | ## |
| 44 | 5.36 | 295.2285 | C_18_H_32_O_3_ | 13S-hydroxyoctadecadienoic acid | HMDB0004667 | ↑* | - | - | ## |
| 45 | 5.45 | 452.2798 | C_28_H_39_NO_4_ | Sambutoxin | HMDB0041085 | ↑* | # | # | # |
| 46 | 5.74 | 452.28 | C_21_H_44_NO_7_P | LysoPE(0:0/16:0) | HMDB0011473 | ↑* | - | - | - |

Note: ↑:The content of the model group was higher than that of the blank group.

↓:The content of the model group was lower than that of the blank group.

*: Compared with the blank group, the content of markers in the model group changed significantly,p<0.05.

**: Compared with the blank group, the content of markers in the model group changed very significantly,p<0.01.

#:Compared with the control group, the content of markers changed significantly,p<0.05.

##:Compared with the control group, the content of markers changed very significantly,p<0.01.

-:There was no significant change in the content of biomarkers.
